# Vitamin D and K Supplementation Is Associated with Changes in the Methylation Profile of U266-Multiple Myeloma Cells, Influencing the Proliferative Potential and Resistance to Bortezomib

**DOI:** 10.3390/nu16010142

**Published:** 2023-12-31

**Authors:** Karolina Łuczkowska, Piotr Kulig, Bartłomiej Baumert, Bogusław Machaliński

**Affiliations:** 1 Department of General Pathology, Pomeranian Medical University, 70-111 Szczecin, Poland; piotrkulig@interia.eu (P.K.); boguslaw.machalinski@pum.edu.pl (B.M.); 2 Department of Hematology and Transplantology, Pomeranian Medical University, 71-252 Szczecin, Poland

**Keywords:** multiple myeloma, vitamin D, bortezomib, drug resistance

## Abstract

Multiple myeloma (MM) is a plasma cell malignancy that, despite recent advances in therapy, continues to pose a major challenge to hematologists. Currently, different classes of drugs are applied to treat MM, among others, proteasome inhibitors, immunomodulatory drugs, and monoclonal antibodies. Most of them participate in an interplay with the immune system, hijacking its effector functions and redirecting them to anti-MM activity. Therefore, adjuvant therapies boosting the immune system may be potentially beneficial in MM therapy. Vitamin D (VD) and vitamin K (VK) have multiple so called “non-classical” actions. They exhibit various anti-inflammatory and anti-cancer properties. In this paper, we investigated the influence of VD and VK on epigenetic alterations associated with the proliferative potential of MM cells and the development of BTZ resistance. Our results showed that the development of BTZ resistance is associated with a global decrease in DNA methylation. On the contrary, both control MM cells and BTZ-resistant MM cells exposed to VD alone and to the combination of VD and VK exhibit a global increase in methylation. In conclusion, VD and VK in vitro have the potential to induce epigenetic changes that reduce the proliferative potential of plasma cells and may at least partially prevent the development of resistance to BTZ. However, further ex vivo and in vivo studies are needed to confirm the results and introduce new supplementation recommendations as part of adjuvant therapy.

## 1. Introduction

Multiple myeloma (MM) is an incurable plasma cell malignancy; nevertheless, the implementation of novel therapies has significantly improved patient outcomes. One of the turning points in MM therapy was the implementation of bortezomib (BTZ), a potent anti-MM drug that could be included in numerous chemotherapy regimens. It acts as a proteasome inhibitor. More precisely, BTZ reversibly blocks the 26S proteasome unit. Blocking this molecular pathway inhibits the degradation and thus the turnover and metabolism of proteins, events that are essential for cell proliferation and survival, ultimately leading to growth inhibition and apoptosis [1]. In addition to the main mechanism of action, there are also less-known downstream mechanisms that interfere with cellular metabolism. It has been shown that BTZ can induce epigenetic changes in cells exposed to the drug. Importantly, epigenetic alterations appear to be responsible for some adverse effects and may also contribute to the occurrence of BTZ resistance. For instance, BTZ can induce complex epigenetic alterations in nerve cells, including changes in methylation profile as well as in miRNAs expression, which subsequently regulate the expression of their target genes. All these changes were demonstrated to contribute to the development of BTZ-induced peripheral neuropathy in MM patients [2]. Moreover, in the neuroblastoma cell line, BTZ has the propensity to induce aberrant expression of miRNAs and alter miRNA–mRNA interactions in nerve cells, enhancing apoptosis and neuronal death as well as inhibiting neurogenesis [3]. In addition, global changes in the methylation profile were associated with the development of BTZ resistance in a human neuroblastoma cell line [4].

Vitamin D (VD) is a prohormone that, after hydroxylation in the liver and kidneys, acts through the vitamin D receptor (VDR). VDR is located in the nucleus and regulates the expression of multiple genes when activated [5]. It is very well-known that VD actions are tightly bound to calcium metabolism and bone health [6]. However, the VDR is located not only in tissues related to calcium metabolism and bone health, such as the intestines, bones, and kidneys, but also in numerous other cell types. The location of VDR implies that VD has under its influence multiple processes in many different tissues. In addition to the main mechanism of action via VDR, there are also membrane receptors responsible for non-genomic mechanisms. Their activation results in the activation of various downstream activities such as the opening of ion channels and subsequent generation of signaling molecules and second messengers [7]. Several studies suggest that VD itself has no significant potential to directly induce epigenetic changes [8,9]. Nevertheless, it was demonstrated by Chauss and colleagues that VD can exert epigenetic changes in helper T cells [10]. In addition, our team demonstrated the upregulation of genes from the SNORD family, which is associated with, among others, methylation, in the U266 MM cell line treated with different combinations of BTZ, VD, and vitamin K (VK) [11]. Therefore, we decided to investigate epigenetic changes and their role in the development of BTZ resistance. VD research is currently experiencing its renaissance.

There is a focus on its so-called non-classical actions, i.e., not directly related to bone health, phosphate, and calcium metabolism. Its influence on both the adaptive and innate immune response is particularly important [12]. Modulation of the immune system results in plenty of anti-cancer entities. VD has been shown to induce differentiation and decrease the proliferation of malignant cells [5]. Its anti-tumor activity was demonstrated for solid tumors, for instance, by enhancing the response to chemotherapy [13], as well as hematological malignancies, including MM [14,15]. The exact role of VD in MM has been reviewed by our team elsewhere [16]. On top of VD, VK (particularly the K2 MK7 isoform) was demonstrated to exhibit various anti-cancer effects [17], which were also apparent in hematological malignancies [18,19] including MM [20]. Although, as mentioned above, VK possesses anti-cancer properties, these are not as prominent as in VD. In fact, VK is rarely supplemented alone in different settings than coagulation abnormalities due to VK deficiency or VK antagonists. Nevertheless, there is evidence that VD and VK act synergistically, especially in terms of bone and cardiovascular health. Moreover, VK prevents calcification [21,22,23,24]. This provides the rationale for supplementation with both vitamins together, rather than considering VK as an independent anti-MM agent. Therefore, it may be hypothesized that those vitamins may enhance their biological effects when administered together in MM. If their efficacy was confirmed in clinical trials, these vitamins could in the future be recommended for MM patients as an adjuvant therapy. We recently demonstrated that VD and VK have the potential to affect the proliferative potential of MM cells in vitro. Moreover, we found that one of the underlying mechanisms is mediated by genes belonging to the SNORD family, which are associated with epigenetic alterations [11]. After establishing that VD and VK treatment could induce epigenetic modifications in MM cells, we decided to further explore this area.

In our previous paper, we thoroughly described the proliferation analysis. Briefly, after the first treatment with BTZ, a reduction in proliferation of 70.06% was noted compared to control cells (*p* < 0.0001). After the second treatment with BTZ, there was a decrease in proliferation by 24.12% (*p* < 0.0001). The third treatment resulted in the development of a BTZ-resistant phenotype. The addition of 25(OH)D3 resulted in a decrease in proliferation by more than 20%. Similar effects were observed when VK was added. In non-BTZ-resistant cells, vitamins exhibited synergic effects with BTZ. What is particularly important is that vitamins reduced the proliferation of BTZ-resistant U266 MM cells. To see a detailed analysis, please refer to our previous paper [11].

The aim of this study was to further investigate the role of VD and VK2 MK7 in MM. We wanted to assess the effect of VD and VK on epigenetic alterations associated with the proliferative potential of MM cells and the development of BTZ resistance. Furthermore, we intend to provide a molecular background for subsequent clinical trials. The results are presented by dividing them into three sections. The first shows the influence of methylation on the development of BTZ resistance, the second shows the influence of VD and VK on the alteration of the methylation profile in cancer cells not resistant to BTZ, and the third presents the methylation profile after the action of VD and VK on cancer cells with a BTZ-resistant phenotype.

## 2. Materials and Methods

### 2.1. Cell Culture and the Course of the Experiment

The human multiple myeloma cell line U266 (ATCC, Manassas, VA, USA) was used in this study. U266 cells were cultured according to the manufacturer’s instructions with RPMI-1640 medium (ATCC, Manassas, VA, USA, cat no. 30-2001) modified to contain 2 mM L-glutamine, 10 mM HEPES, 1 mM sodium pyruvate, 4500 mg/L glucose, and 1500 mg/L sodium bicarbonate. A complete growth medium was prepared by adding fetal bovine serum to the basal medium to a final concentration of 15%. The medium was changed every three days. U266 cells were treated three times with BTZ at 2.75 nM (Cell Signaling Technology, Danvers, MA, USA) and/or 25(OH)D3 (VD) (10^−6^ M) (Sigma-Aldrich, Saint Louis, MO, USA), and/or K2MK7 (VK) (10^−5^ M) (Sigma-Aldrich, Saint Louis, MO, USA) for 24 h with 10-day intervals between treatments. The dose of BTZ was established in our previous articles [11,25] and causes over 50% of cell death after the first treatment. The third treatment results in a BTZ-resistant cell phenotype. In addition, the effectiveness of the BTZ-resistant cell line derivation scheme was confirmed on the SH-SY5Y neuroblastoma cell line in our previous article [4]. Vitamin concentrations were established in our previous article [11]. DNA was isolated from the cells after each treatment and portions of the cells were left to proliferate further in a medium without BTZ and vitamins. In the experiment, four study groups (D—treated with vitamin 25(OH)D3; BTZ—treated with bortezomib; DK—treated simultaneously with vitamin 25(OH)D3 and K2MK7; BTZ_DK—treated simultaneously with bortezomib, vitamin 25(OH)D3, and K2MK7); and a control group were distinguished.

### 2.2. DNA Extraction and Bisulfate Conversion

DNA was isolated from three separate cell incubations for all groups. PureLink Genomic DNA Mini Kit (Thermo Fisher, Waltham, MA, USA) was used for DNA isolation following the manufacturer’s instructions. The concentration and quality of the isolated DNA were measured using TapeStation 4510 (Agilent Technologies, Santa Clara, CA, USA) and the Genomic DNA ScreenTape kit (Agilent Technologies, Santa Clara, CA, USA). All samples showed DINs ≥ 9, which confirms the high quality of the genetic material. The bisulfate conversion was performed using the EZ DNA Methylation-Gold Kit (Zymo Research, Irvine, CA, USA) according to the manufacturer’s instructions. In total, 500 ng of DNA from each sample was used for conversion.

### 2.3. Methylation Arrays

An Infinium MethylationEPIC v2.0 BeadChip Kit, human (Illumina, San Diego, CA, USA) was used to analyze the methylation profile. Methylation arrays were performed in triplicate for each group included in the experiment (n = 3). Methylation arrays were made strictly in accordance with the manufacturer’s instructions. In short, after bisulfate conversion, the genetic material was amplified for 24 h. The DNA was then fragmented and precipitated, allowing the material to hybridize with the BeadChips array. Hybridization was carried out for 20 h at 48 °C. The next steps were to wash out non-hybridized and non-specifically hybridized DNA from the BeadChips, add labeled nucleotides to extend the primers hybridized with the sample, and stain them. The NextSeq550 instrument (Illumina, San Diego, CA, USA) was used to scan the arrays.

### 2.4. Bioinformatics Analysis of Genome-Wide Methylation

Screening for methylation changes was performed using the Illumina Infinium Methylation EPIC Beadchip array (Illumina, San Diego, CA, USA). The analyses were carried out in the R programming environment with the relevant Bioconductor libraries. We presented a detailed description of bioinformatics analyses in our previous article [25].

### 2.5. Gene Expression Analysis by qRT-PCR

The qRT-PCR method was used to analyze the expression of selected genes (*ARHGAP26*, *MYH10*, *PMP2*, *RFX8*, *BAMBI*, *CLEC12b*). The main criterion for selecting the above genes was their significant variability in the methylation level between the studied groups and their possible involvement in the discussed processes. Primers were designed using the BLAST program (Table 1). The qRT-PCR method consists of two stages. The first one requires performing the mRNA reverse transcription process (0.1 µg) using the First Strand cDNA Synthesis Kit (Thermo Fisher Scientific, Waltham, MA, USA), and the second one is a qPCR reaction using the SYBR Green PCR Master Mix kit (Bio-Rad, Hercules, CA, USA). The GAPDH gene was used as an endogenous control gene. qPCR reactions were performed on a Bio-Rad CFX96 Real-Time PCR Detection System (Bio-Rad Inc., Hercules, CA, USA).

## 3. Results

### 3.1. DNA Methylation Changes

Analysis of global changes in the methylation profile was performed after each treatment in each group. However, the article presents the results obtained after the last, third, treatment. After the first treatment with BTZ and/or vitamins, we did not obtain any significant changes in methylation. After the second treatment, there were slight changes in the groups: BTZ_DK_2 vs. BTZ_2 (21 genes with altered methylation); D_2 vs. Control_2 (128 genes with altered methylation); DK_2 vs. Control_2 (150 genes with altered methylation). The results of the bioinformatics analysis after the second treatment are included in the Supplementary Material (Figures S1–S3).

#### 3.1.1. DNA Methylation Changes Associated with the Development of Resistance of U266 Myeloma Cells to BTZ

First, we investigated the epigenetic mechanisms involved in the development of BTZ resistance. We compared control MM cells with BTZ-resistant plasma cells and performed a bioinformatics analysis that revealed epigenetic alterations and mechanisms underlying the development of resistance to BTZ.

**Figure 1:**
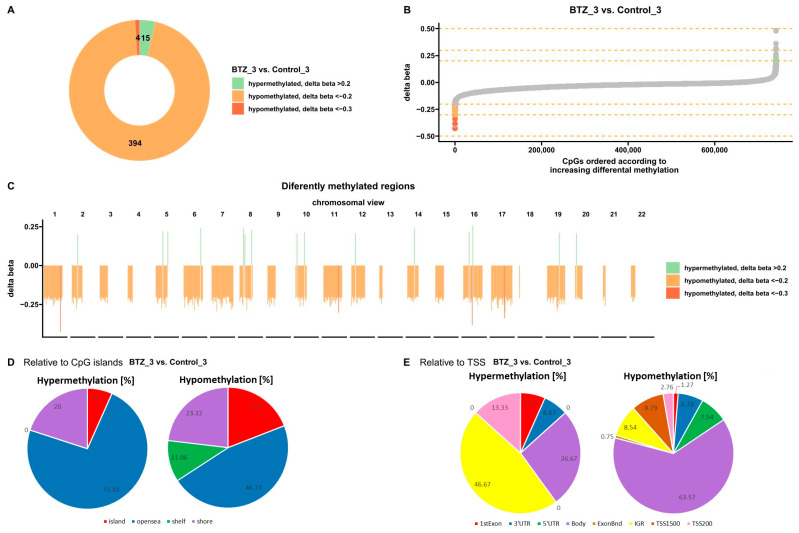
Methylation profile after three consecutive BTZ treatments of U266 myeloma cells. (**A**,**B**) charts show differences in methylation levels in BTZ-treated U266 cells relative to untreated control cells (*p* < 0.05). Orange represents hypomethylation and green represents hypermethylation (**C**) View of changes in the level of methylation in individual chromosomes. Orange represents hypomethylation and green represents hypermethylation (*p* < 0.05). (**D**) Classification of differentially methylated sites in the genome according to their location relative to the CpG islands (*p* < 0.05). (**E**) Classification of differentially methylated sites in the genome according to their location relative to the transcription start site (TSS) (*p* < 0.05). (D—treated with vitamin 25(OH)D3; BTZ—treated with bortezomib; DK—treated simultaneously with vitamin 25(OH)D3 and K2MK7; BTZ_DK—treated simultaneously with bortezomib, vitamin 25(OH)D3, and K2MK7; Control—control group; 3—third incubation).

Bioinformatics analysis showed 413 sites with altered methylation in cells treated three times with BTZ compared to untreated control cells (Figure 1A). Delta beta values were calculated according to the normalized ratios of probe fluorescence intensity between methylated and unmethylated signals (value 0 = fully unmethylated, 1 = fully methylated).

The analysis identified 398 hypomethylated and 15 hypermethylated sites (Figure 1A,B). In addition, these differences are shown on each chromosome (Figure 1C). Hypermethylated sites are marked in green and hypomethylated sites in orange. Further analysis allowed the classification of the altered sites according to the location of the CpG islands (regions with a high concentration of phosphate-linked cytosine–guanine pairs found in many gene promoters) (Figure 1D) and the transcription start site (TSS) (Figure 1E). Changes in methylation levels were observed in the following regions of the genome: open sea (isolated CpG sites in the genome that do not have a specific designation), shelf (regions 2–4 kb from CpG islands), shore (regions 0–2 kb from CpG islands). Most of the altered sites, both hypo- and hypermethylated, were found in the open sea. Overall, 19.1% of hypomethylated sites and 6.67% of hypermethylated sites were observed in CpG islands. In addition, no hypermethylated shelf changes were observed. An increased level of methylation in relation to TSS was mainly observed in the IGR (intergenic region) and decreased in the body. More importantly, no hypermethylation was observed in TSS1500. In contrast, hypomethylation sites were observed in both TSS1500 and TSS200 (Figure 1E).

**Figure 2:**
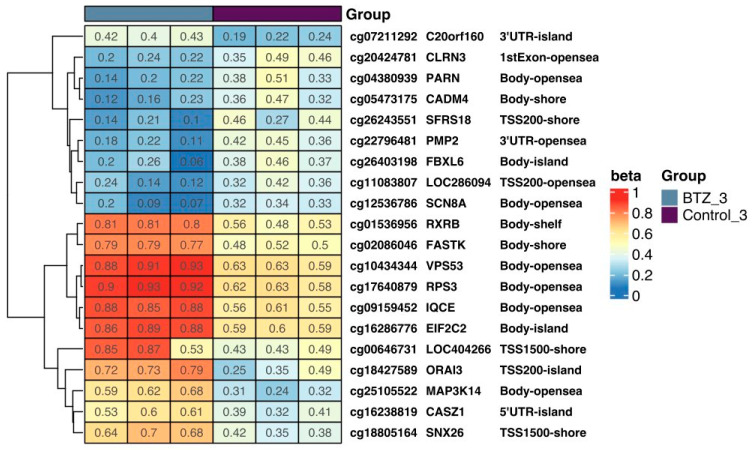
The heat map shows the methylation level of selected genes in control cells and cells treated three times with BTZ. Methylation results are presented as beta values where 1 means full methylation (red) and 0 means no methylation (blue) (*p* < 0.05). In addition, gene symbols and methylation locations are marked on the heat map. Heatmap results were visualized using the R library ComplexHeatmap, version 2.13.1.

Methylation levels in selected genes in control cells and cells treated three times with BTZ are shown in Figure 2 as a heatmap. The most important observations are the hypomethylation of the *FBXL6*, *CLRN3*, and *PMP2* genes in BTZ-treated cells relative to control cells. Their high expression is associated with tumor progression and poor prognosis in oncological patients [26]. At the same time, hypermethylation of the VPS53 gene was observed (Figure 2), which enhances the process of apoptosis and autophagy.

##### *Gene Set Enrichment Analysis* (*GSEA*)

**Figure 3:**
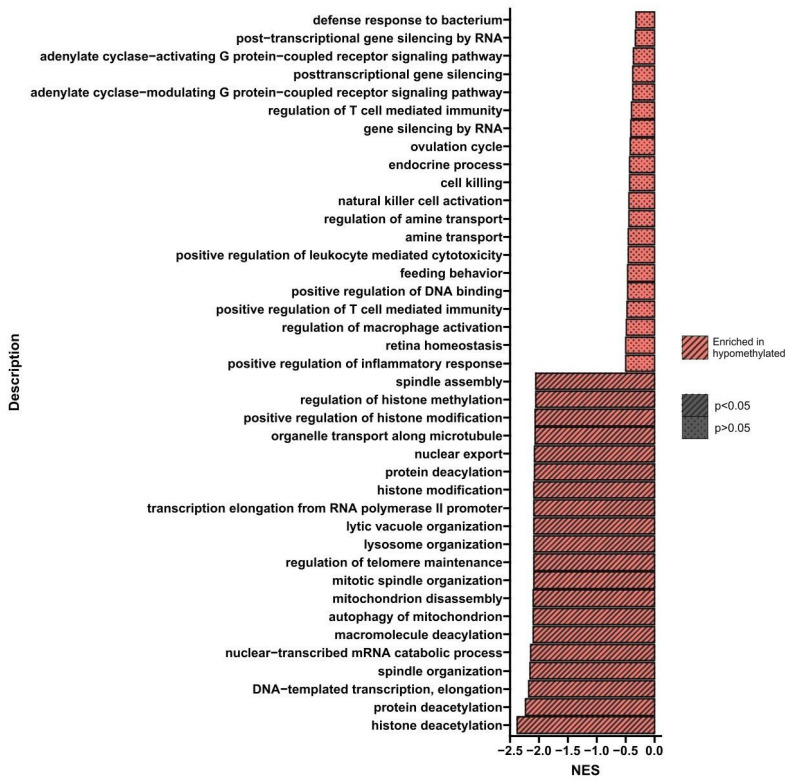
GSEA shows biological processes whose genes showed a change in methylation levels in U266 cells treated three times with BTZ compared to control cells. Red indicates hypomethylation and green indicates hypermethylation. NES—normalized enrichment score.

GSEA is a bioinformatics tool that enables the isolation of groups of genes related to a specific biological process or signaling pathway. Figure 3 shows the processes whose genes altered methylation levels in cells treated three times with BTZ compared to control cells. Only processes whose genes were hypomethylated were identified (*p* < 0.05). The most important processes seem to be the regulation of histone methylation, histone modification, protein deacylation, regulation of telomere maintenance, DNA-templated transcription, and elongation.

The development of BTZ resistance is associated with alterations in epigenetic mechanisms. We demonstrated above that BTZ-resistant plasma cells exhibit a different methylation profile than control MM cells. Taken together, the development of BTZ resistance is associated with the global hypomethylation of MM cells.

#### 3.1.2. Effect of VD on DNA Methylation Changes in U266 Myeloma Cells

Subsequently, we intended to explore if VD has the proclivity to induce epigenetic changes in control MM cells. Therefore, we investigated changes in the global methylation profile in control MM cells. In addition, we analyzed how exposure to VD affected various cellular processes and identified the genes whose methylation profile was most altered.

**Figure 4:**
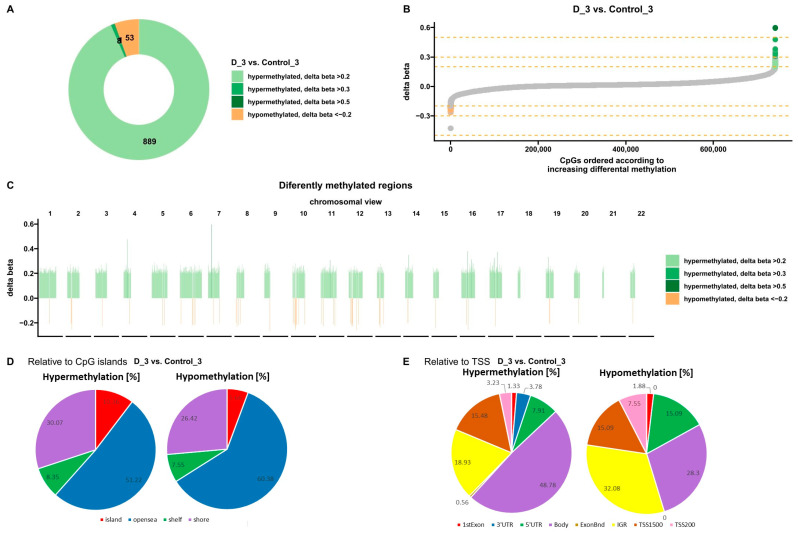
Methylation profile after three VD treatments of U266 myeloma cells. (**A**,**B**) Charts show differences in methylation levels in U266 cells treated with VD relative to untreated control cells (*p* < 0.05). Orange represents hypomethylation and green represents hypermethylation (**C**) View of changes in the level of methylation in individual chromosomes. Orange represents hypomethylation and green represents hypermethylation (*p* < 0.05). (**D**) Classification of differentially methylated sites in the genome according to their location relative to the CpG islands (*p* < 0.05). (**E**) Classification of differentially methylated sites in the genome according to their location relative to the transcription start site (TSS) (*p* < 0.05). (D—treated with vitamin 25(OH)D3; BTZ—treated with bortezomib; DK—treated simultaneously with vitamin 25(OH)D3 and K2MK7; BTZ_DK—treated simultaneously with bortezomib, vitamin 25(OH)D3, and K2MK7; Control—control group; 3—third incubation).

Bioinformatics analysis showed 950 sites with altered methylation in cells treated three times with VD compared to untreated control cells (Figure 4A). The analysis identified 53 hypomethylated sites and 897 hypermethylated sites (Figure 4A,B). In addition, these differences are shown on each chromosome (Figure 4C). Hypermethylated sites are marked in green and hypomethylated in orange. Further analysis allowed the classification of altered sites according to the location of the CpG islands (Figure 4D) and the transcription start site (TSS) (Figure 4E).

Most altered sites, both hypo- and hypermethylated, were found in the open sea. In total, 5.66% of hypomethylated sites and 10.36% of hypermethylated sites were observed in CpG islands. Both increases and decreases in methylation relative to TSS were mainly observed in the IGR and body (the entire gene from the transcription start site to the end of the transcript) (Figure 4E). More importantly, no hypomethylation was observed in the 3′UTR, a regulatory region that can affect the expression of many genes.

**Figure 5:**
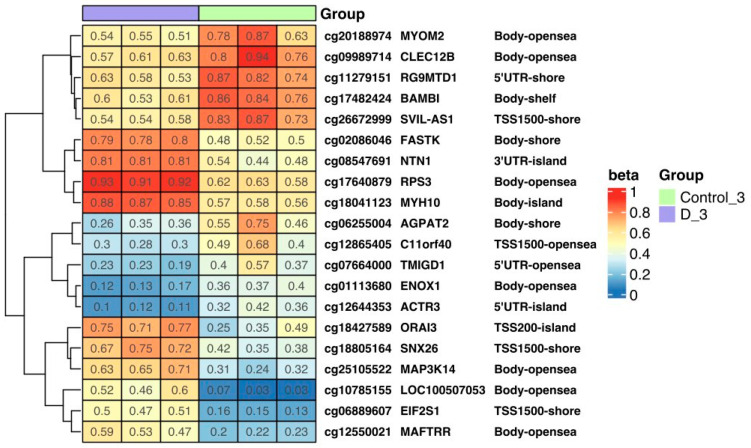
The heat map shows the methylation level of selected genes in control cells and triple-VD-treated cells. Methylation results are presented as beta values where 1 means full methylation (red) and 0 means no methylation (blue) (*p* < 0.05). In addition, gene symbols and methylation locations are marked on the heat map. (D—treated with vitamin 25(OH)D3; BTZ—treated with bortezomib; DK—treated simultaneously with vitamin 25(OH)D3 and K2MK7; BTZ_DK—treated simultaneously with bortezomib, vitamin 25(OH)D3, and K2MK7; Control—control group; 3—third incubation). Heatmap results were visualized using the R library ComplexHeatmap.

Methylation levels in selected genes in control cells and triple-VD-treated cells are shown in Figure 5 as a heatmap. The most important observations are the hypomethylation of the *CLEC12B* and *BAMBI* genes, which are responsible for inhibiting the proliferation of cancer cells. The study also observed an increase in the methylation levels of genes (NTN1, MYH10) involved in tumor development, progression, proliferation, and migration.

##### *Gene Set Enrichment Analysis* (*GSEA*)

**Figure 6:**
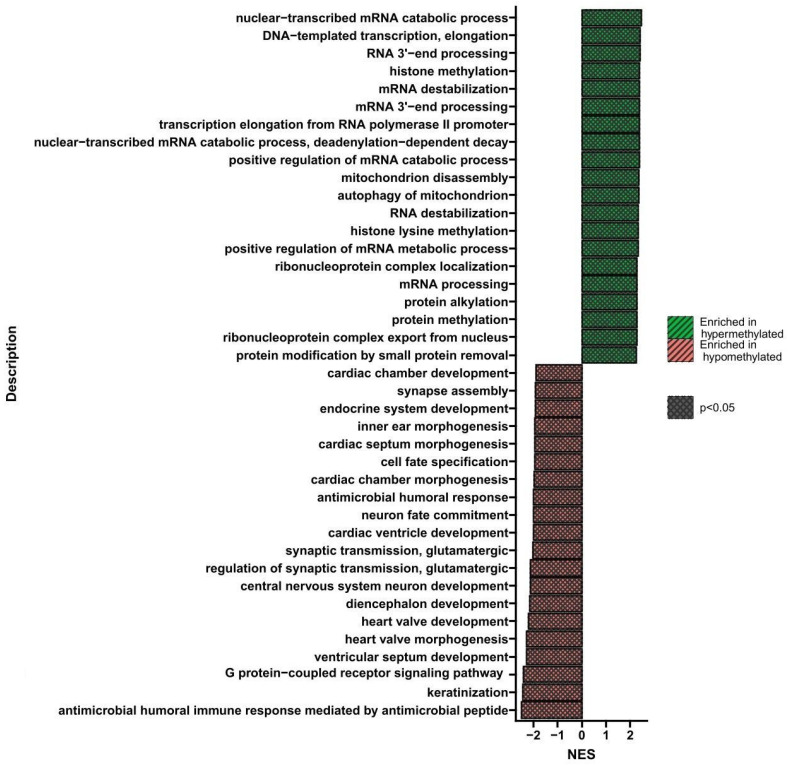
GSEA shows biological processes whose genes showed a change in methylation levels in U266 cells treated three times with VD compared to control cells. Red indicates hypomethylation and green indicates hypermethylation. NES—normalized enrichment score.

Figure 6 shows the processes whose genes altered methylation levels in triple-VD-treated cells compared to control cells. Hypo- and hypermethylated genes were identified and assigned to the specific processes they regulate (*p* < 0.05). The most important hypermethylated gene processes are histone methylation, mRNA destabilization, and protein methylation.

VD significantly affects the methylation profile in MM cells. Both hypo- and hypermethylation were observed. More precisely, we identified genes in which various regions were predominantly hypermethylated, yet in some of them, hypomethylation was also observed. Similarly, in multiple cellular processes, we revealed hyper- and hypomethylation. Nevertheless, in summary, co-culturing with VD is associated with a global increase in methylation.

#### 3.1.3. Effect of VD and VK on DNA Methylation Changes in U266 Myeloma Cells

Next, we evaluated the impact of the combination of VD and VK on the methylation profile in control MM cells.

**Figure 7:**
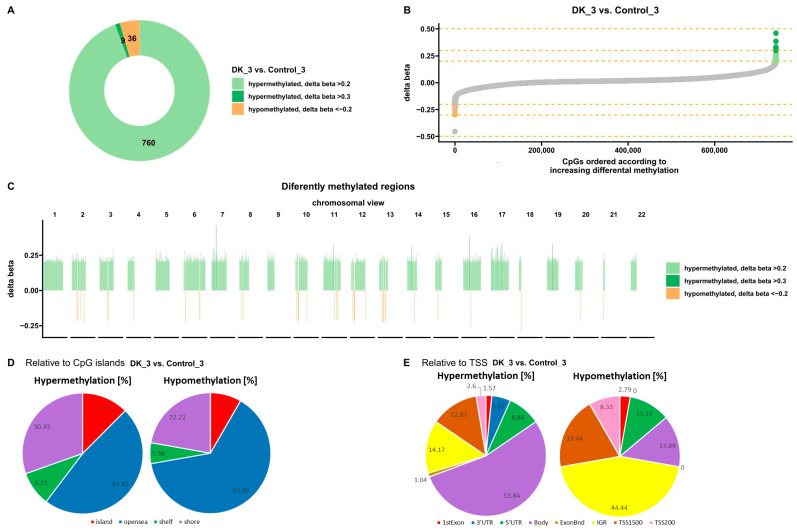
Methylation profile after three VD and VK treatments of U266 myeloma cells. (**A**,**B**) Charts show differences in methylation levels in U266 cells treated with VD and VK compared to untreated control cells (*p* < 0.05). Orange represents hypomethylation and green represents hypermethylation (**C**) View of changes in the level of methylation in individual chromosomes. Orange represents hypomethylation and green represents hypermethylation (*p* < 0.05). (**D**) Classification of differentially methylated sites in the genome according to their location relative to the CpG islands (*p* < 0.05). (**E**) Classification of differentially methylated sites in the genome according to their location relative to the transcription start site (TSS) (*p* < 0.05). (D—treated with vitamin 25(OH)D3; BTZ—treated with bortezomib; DK—treated simultaneously with vitamin 25(OH)D3 and K2MK7; BTZ_DK—treated simultaneously with bortezomib, vitamin 25(OH)D3, and K2MK7; Control—control group; 3—third incubation).

Bioinformatics analysis showed 805 sites with altered methylation in cells treated three times with VD and VK compared to untreated control cells (Figure 7A). The analysis identified 36 hypomethylated sites and 769 hypermethylated sites (Figure 7A,B). In addition, these differences are shown on each chromosome (Figure 7C). Hypermethylated sites are marked in green and hypomethylated in orange. Further analysis allowed the classification of the altered sites according to the location of the CpG islands (Figure 7D) and the transcription start site (TSS) (Figure 7E). Most altered sites, both hypo- and hypermethylated, were found in the open sea. In addition, 8.33% of hypomethylated sites and 12.48% of hypermethylated sites were observed in CpG islands. Regarding TSS, an increase in the level of methylation was observed mainly in the body, and a decrease in IGR (Figure 7E). More importantly, no hypomethylation was observed in the 3′UTR.

**Figure 8:**
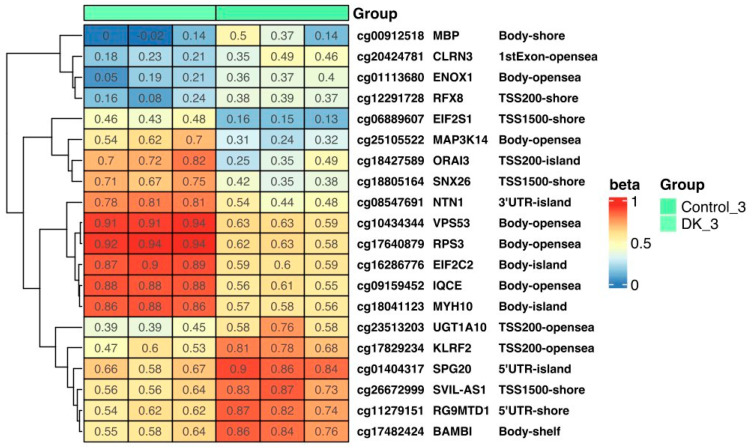
The heat map shows the methylation level of selected genes in control cells and triple-VD- and -VK-treated cells. Methylation results are presented as beta values where 1 means full methylation (red) and 0 means no methylation (blue) (*p* < 0.05). In addition, gene symbols and methylation locations are marked on the heat map. Heatmap results were visualized using the R library ComplexHeatmap.

Methylation levels in selected genes in control cells and triple-VD- and -VK-treated cells are shown in Figure 8 as a heatmap. The most important finding is the hypomethylation of the *RFX8* gene in cells treated three times with VD and VK. This gene is thought to be involved in the regulation of transcription by RNA polymerase II and thus, in the regulation of various processes [27]. Moreover, hypermethylation of the *NTN 1* gene was noted, whose epigenetic regulation was associated with the development of colorectal cancer [28].

**Figure 9:**
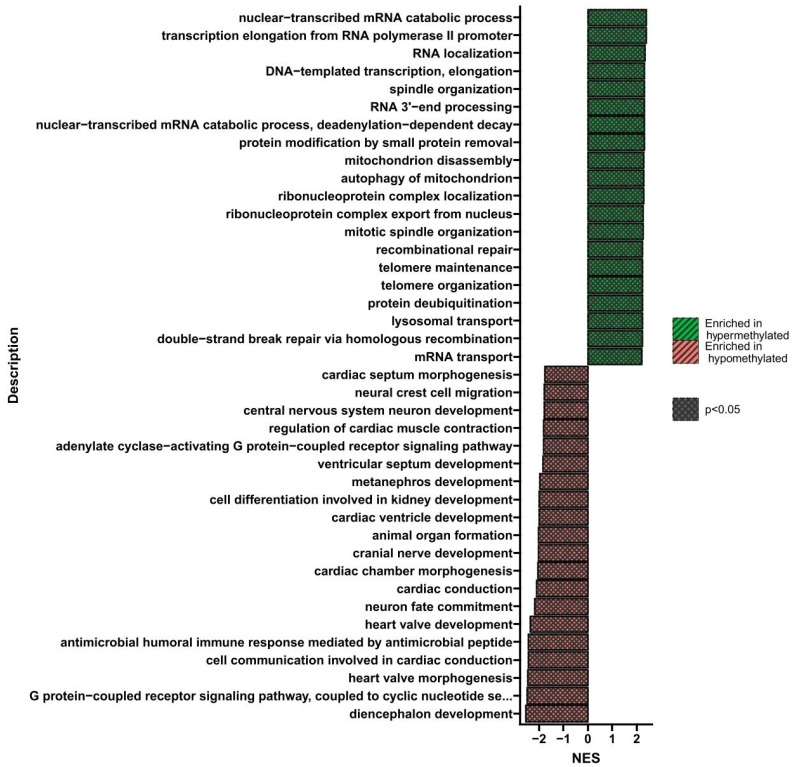
GSEA shows the biological processes whose genes showed a change in methylation levels in U266 cells treated three times with VD and VK compared to control cells. Red indicates hypomethylation and green indicates hypermethylation. NES—normalized enrichment score.

Figure 9 shows the processes whose genes had altered methylation levels in triple-VD- and -VK-treated cells compared to control cells. Hypo- and hypermethylated genes have been identified and assigned to the individual processes they regulate (*p* < 0.05). Hypermethylated gene processes are RNA localization, DNA-templated transcription, elongation, and telomere organization.

MM cells exposed to the combination of VD and VK exhibited a similar methylation profile to control cells co-cultured with VD alone. Similarly, we identified genes in which various regions were predominantly hypermethylated; however, hypomethylation was also observed in some of them. Moreover, we detected both hyper- and hypomethylation in various cellular processes. In conclusion, similarly to VD alone, the combination of VD and VK is associated with a global increase in methylation.

#### 3.1.4. DNA Methylation Changes Induced by VD and VK in U266 Myeloma Cells in a BTZ-Resistant Phenotype

We established that VD alone and in combination with VK has the potential to induce epigenetic alterations in control MM cells. Thus, we hypothesized that these vitamins may also affect the methylation profile in MM cells exhibiting a BTZ-resistant phenotype. Therefore, we conducted a similar analysis to explore the impact of both VD and VK on BTZ-resistant MM cells.

**Figure 10:**
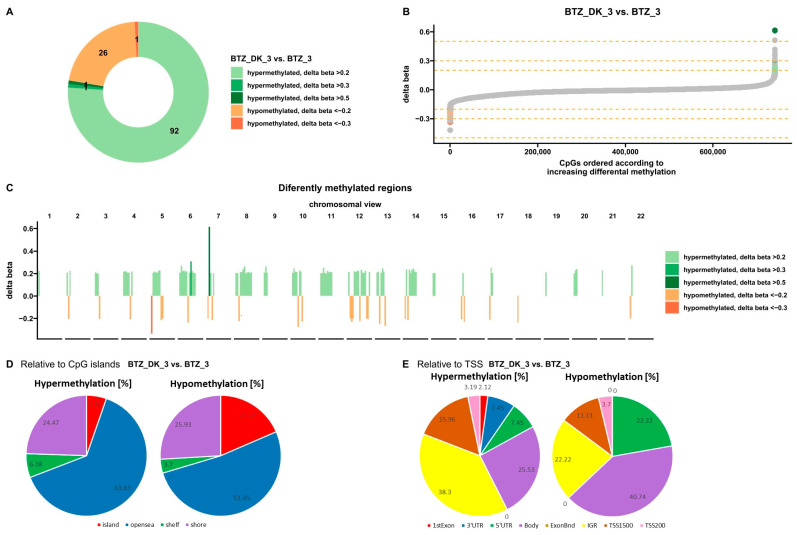
Methylation profile after three subsequent BTZ, VD, and VK treatments of U266 myeloma cells. (**A**,**B**) Charts show differences in methylation levels in U266 cells treated with BTZ, VD, and VK relative to cells treated with BTZ alone (*p* < 0.05). Orange represents hypomethylation and green represents hypermethylation (**C**) View of changes in the level of methylation in individual chromosomes. Orange represents hypomethylation and green represents hypermethylation (*p* < 0.05). (**D**) Classification of differentially methylated sites in the genome according to their location relative to the CpG islands (*p* < 0.05). (**E**) Classification of differentially methylated sites in the genome according to their location relative to the transcription start site (TSS) (*p* < 0.05). (D—treated with vitamin 25(OH)D3; BTZ—treated with bortezomib; DK—treated simultaneously with vitamin 25(OH)D3 and K2MK7; BTZ_DK—treated simultaneously with bortezomib, vitamin 25(OH)D3, and K2MK7; Control—control group; 3—third incubation).

Bioinformatics analysis showed 121 sites with altered methylation in cells treated three times simultaneously with BTZ, VD, and VK compared to cells treated three times with BTZ alone (Figure 10A). The analysis identified 27 hypomethylated sites and 94 hypermethylated sites (Figure 10A,B). In addition, these differences are shown on each chromosome (Figure 10C). Hypermethylated sites are marked in green and hypomethylated in orange. Further analysis allowed the classification of the altered sites according to the location of the CpG islands (Figure 10D) and the transcription start site (TSS) (Figure 10E). Most altered sites, both hypo- and hypermethylated, were found in the open sea. Overall, 18.52% of hypomethylated sites and 5.32% of hypermethylated sites were observed in CpG islands. Anincreased level of methylation in relation to TSS was mainly observed in the IGR and decreased in the body. More importantly, no hypomethylation was observed in the 3′UTR (Figure 10E).

**Figure 11:**
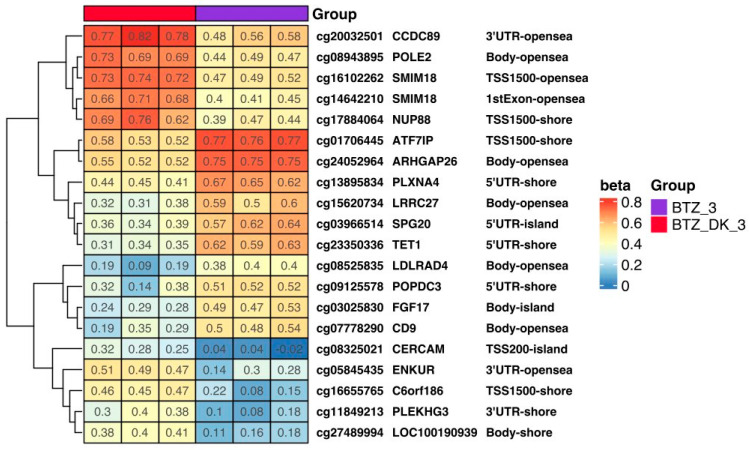
The heat map shows the methylation level of selected genes in cells treated three times with BTZ alone (BTZ_3) and with BTZ, VD, and VK (BTZ_DK_3). Methylation results are presented as beta values, where 1 means full methylation (red) and 0 means no methylation (blue) (*p* < 0.05). In addition, gene symbols and methylation locations are marked on the heat map. (D—treated with vitamin 25(OH)D3; BTZ—treated with bortezomib; DK—treated simultaneously with vitamin 25(OH)D3 and K2MK7; BTZ_DK—treated simultaneously with bortezomib, vitamin 25(OH)D3, and K2MK7; Control—control group; 3—third incubation). Heatmap results were visualized using the R library ComplexHeatmap.

Methylation levels in selected genes in cells treated three times with BTZ, VD, and VK (BTZ_DK_3) and in cells treated with BTZ alone cells are shown in Figure 11 as a heatmap. The most important observation is the hypermethylation of the *ARHGAP26* gene in cells treated three times with BTZ, which is involved in tumorigenesis and progression of human cancers [29].

**Figure 12:**
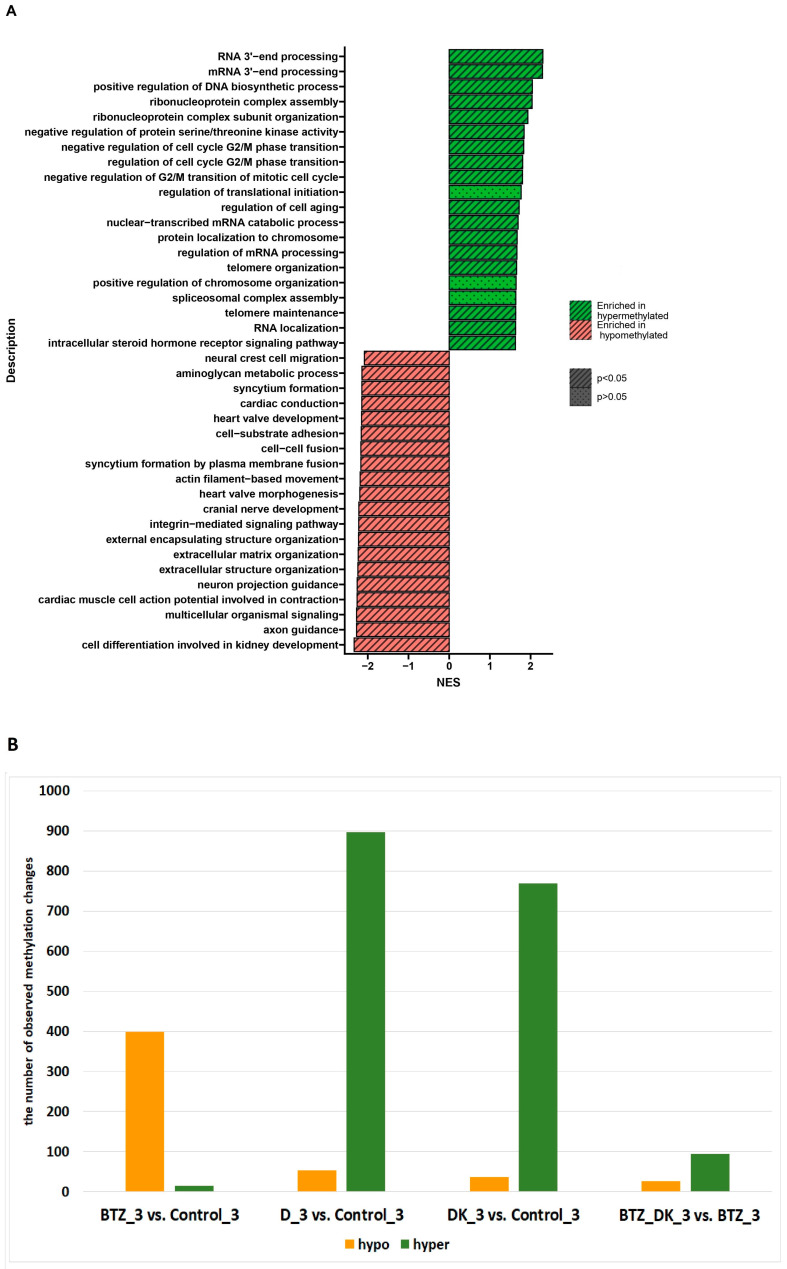
(**A**) GSEA shows biological processes whose genes showed a change in methylation levels in U266 cells treated three times with BTZ, VD, and VK compared to cells treated three times with BTZ alone. Red indicates hypomethylation and green indicates hypermethylation. NES—normalized enrichment score. (**B**) number of observed methylation changes. Orange indicates hypomethylation, green indicates hypermethylation.

Figure 12A shows the processes whose genes altered methylation levels in triple-BTZ-treated cells compared to cells treated three times with BTZ, VD, and VK. Hypo- and hypermethylated genes have been identified and assigned to the individual processes they regulate (*p* < 0.05). The most important hypomethylated gene processes are cell–cell fusion and cell–substrate adhesion and hypermethylated are RNA 3′-end processing, mRNA 3′-end processing, positive regulation of DNA biosynthetic process, and regulation of cell aging.

Figure 12B shows global changes in methylation in control cells in comparison with BTZ_3, D_3, and DK_3 cells as well as in BTZ_3 cells compared with BTZ_DK_3 cells. (D—treated with vitamin 25(OH)D3; BTZ—treated with bortezomib; DK—treated simultaneously with vitamin 25(OH)D3 and K2MK7; BDK—treated simultaneously with bortezomib, vitamin 25(OH)D3, and K2MK7; Control—control group; 3—third incubation) (*p* < 0.05).

The combination of VD and VK is associated with a global increase in methylation in BTZ-resistant MM cells. However, it should be noted that this effect is not as prominent as in control MM cells. Nevertheless, BTZ-resistant cells, when co-cultured with VD alone or with VD and VK, at least temporarily regained sensitivity to BTZ, which is a promising result and may provide a background for future studies.

### 3.2. Expression of Selected Genes

We selected genes (*ARHGAP26*, *MYH10*, *PMP2*, *RFX8*, *BAMBI*, *CLEC12b*) with significant changes in methylation level to validate their expression using qRT-PCR in U266 myeloma cells (Table 2). The selected genes confirmed the actual impact of the methylation level on gene expression (according to the principle of the higher the methylation level, the lower the gene expression and vice versa). From the point of view of the effect of vitamins D and K on the U266 myeloma line, not resistant to BTZ, an important observation is an almost 60% decrease in the expression of the MYH10 gene after three exposures to vitamin D in relation to the control cells and a 73% increase in the expression of the CLEC12b gene. Additionally, after three exposures to both vitamins, a 70% increase in RFX8 gene expression was observed compared to control cells.

However, the most important result is a significant 54% increase in ARHGAP26 gene expression in the BTZ-resistant U266 myeloma cells that were administered vitamin D and K three times compared to BTZ-resistant cells. This result confirms that the lower level of methylation of this gene in BTZ_DK_3 cells (beta value = 0.52 ± 0.01) compared to BTZ_3 (beta value = 0.75 ± 0.00) correlates with a higher level of its expression.

## 4. Discussion

Multiple myeloma remains an incurable disease in most cases. However, the implementation of novel therapies has significantly improved patient outcomes. The underlying background of contemporary treatment strategies relies, among others, upon the modulation of the immune system and enhancing its activity against malignant plasma cells. Thalidomide and its derivatives, named lenalidomide and pomalidomide, belong to the class of immunomodulatory drugs (IMiDs). Their pleiotropic mechanisms of actions reach far beyond the simple degradation of Ikaros and Aiolos, two transcription factors essential for B cells, including plasma cells [30,31]. Another class of drugs that modulate the immune system are monoclonal antibodies. Daratumumab and isatuximab are anti-CD38 monoclonal antibodies that have significantly improved clinical outcomes for patients with MM [32,33]. BTZ and carfilzomib belong to a proteasome inhibitor drug class and block the 26S proteasome unit. Blocking this molecular pathway inhibits the degradation and thus, the turnover and metabolism of proteins that are essential for cell proliferation and survival, ultimately leading to growth inhibition and apoptosis [1,34]. Therefore, a BTZ-based regimen can be considered an anti-plasma cell therapy. A combination of drugs modulating the immune system and “anti-plasma cell” drugs such as BTZ turned out to be a highly effective treatment for MM [35,36,37,38]. Considering the above, the implementation of complementary, adjuvant therapies enhancing the activity of the immune system appears to be a reasonable strategy in the treatment of MM.

VD and VK, with particular emphasis on VD, exhibit multiple so-called “non-classical actions” that are peculiarly associated with the modulation of the immune system [39]. In addition to healthy cells, the presence of VDR has been demonstrated in malignant cells [40]. Therefore, it can be hypothesized that VD may potentially affect them and thus, interfere with cancer growth and progression. Indeed, there is ample evidence that malignant growth is prone to VD-related suppression. Fife at al. investigated the effects of VD on the MDA-MB-435 human breast cancer cell line, the LNCaP human prostate cancer cell line, and the U2OS human osteosarcoma cell line. The study demonstrated that VD inhibits proliferation and induces apoptosis in all three tested cell lines [41].

VD anti-cancer activity is not limited to solid tumors but also affects hematological malignancies. Moreover, what is particularly important, VD affects MM cells, exerting its tumoricidal properties. Although most of the studies published to date have been conducted in vitro on MM cell lines, they have yielded promising and encouraging results. Moreover, human studies already conducted confirm the benefits of implementation of VD in the treatment of MM. For instance, according to Busch et al., maintaining a proper VD concentration in MM patients seems to be of paramount importance, especially alongside IMiD-based treatment regimen. They demonstrated in vitro that VD is a key molecule for restoring and maintaining the effector functions of myeloma-associated macrophages and that VD supplementation in combination with IMiDs can enhance the therapeutic efficacy of anti-CD38 antibodies. However, clinical effectiveness needs to be verified in clinical studies [15]. On top of vitamin D itself, its analogs, such as EB1089, also exhibit anti-MM activity. Most importantly, EB1089 has reduced hypercalcemic effects. Despite the promising results of in vitro studies, VD analogs should be further investigated in clinical trials [42,43,44]. Maintaining proper VD status in MM patients appears to be particularly important. For instance, there is an association between low serum VD status and the prevalence of peripheral neuropathy in the population of MM patients [45]. In addition, according to a study by Wang and colleagues, patients with VD deficiency who were treated with either BTZ or lenalidomide were significantly more likely to develop severe peripheral neuropathy of both motor and sensory types [46]. Furthermore, in a study by Eicher et al., patients with MM without VD deficit who underwent high-dose chemotherapy followed by autologous hematopoietic stem cell transplantation have been shown to have lower overall mortality and longer overall survival and progression-free survival [47]. Nevertheless, it should be noted that studies revealing that low VD status is associated with poorer prognosis in MM or in increase in complications such as peripheral neuropathy, or between appropriate serum VD concertation and good clinical outcome, are correlational studies without any intervention (such as VD supplementation). Therefore, clinical trials are needed to ultimately prove the causative relationship between VD status and better clinical outcomes in MM. Interventional studies and clinical trials should be conducted to further explore this area. Particularly interesting would be a study exploring the relationship between VD serum concentration and gene expression profile, similarly to an interesting study conducted by Donati et al. They demonstrated that VD deficiency indeed affects gene expression and activates stress-protective and pro-survival pathways mediated by NF-κB in classical Hodgkin’s lymphoma [48].

We also investigated the potential role of VD in MM. We have demonstrated that 25(OH)D3 has the potential to inhibit the proliferative potential of MM cells. Furthermore, we have shown that 25(OH)D3 can overcome resistance to BTZ in vitro and that its anti-MM properties are at least partially controlled by epigenetic alterations [11].

We have herein deepened our research and thoroughly investigated the epigenetic mechanisms underlying the anti-MM activity of 25(OH)D3 and its role in the development of BTZ resistance. In this study, we showed that the development of BTZ resistance is associated with global changes in methylation. More precisely, MM cells with a BTZ-resistant phenotype were hypomethylated, mainly in the open sea, compared to control cells. In particular, hypomethylation of the *FBXL6*, *CLRN3*, and *PMP2* genes was observed in BTZ-resistant cells compared to control cells. Their high expression is associated with tumor progression and poor clinical outcomes in oncology patients [26,49]. Since these genes have been found to be hypomethylated, it can be hypothesized that given biological processes controlled by them tend to be more active in comparison to control cells and in this way contribute to the resistance development. In addition, GSEA analysis revealed only processes that were hypomethylated. The most significant were histone methylation, histone modification, and protein diacylation, as well as regulation of telomere maintenance. These results suggest that epigenetic mechanisms are associated with BTZ resistance. Globally, resistance to BTZ is associated with hypomethylation. The role of epigenetic mechanisms in tumor progression and the development of drug resistance has been studied in various malignancies. For instance, hypomethylation in prostate cancer is associated with poorer clinical outcomes and more aggressive metastatic disease [50]. Moreover, according to a systematic review by Zelic et al., global DNA methylation levels were consistently lower in prostate cancer tissue compared to healthy prostate tissue. At the same time, methylation levels were lower in more aggressive tumors [51]. Alterations in methylation have also been studied in MM. The results of the study conducted by Sieve and colleagues revealed that global loss of methylation may be a hallmark of progressive disease with poorer clinical outcomes. They found that an extremely high overlap of hypomethylated genes was associated with poorer survival. Interestingly, most of the hypomethylated genes were outside the CpG islands [52]. Moreover, it has been shown that global changes in methylation can play a significant role in myelomagenesis and promote the transition from monoclonal gammopathy of unknown significance (MGUS) to fully symptomatic MM [53]. We have demonstrated in our previous study that incubation with VD or with both VD and VK has the potential to reduce the proliferation of U266 MM cells, including cells exhibiting a BTZ-resistant phenotype [11]. Consistent with our results in this study, the reduced proliferative potential is also reflected in the altered methylation profile. MM cells incubated with VD or with both VD and VK are globally hypermethylated. Consistently with the overall methylation profile, we observed hypermethylation of genes involved in cancer development and progression. For instance, in control U266 MM cells incubated with VD only, an increase in the methylation levels of the *NTN1* and *MYH10* genes was observed. These genes are involved in the development, progression, and proliferation of tumors in a variety of cancers, including hematological malignancies. For instance, Huang et al. demonstrated that *NTN1* expression was upregulated in B-ALL patients with high and intermediate risk. Therefore, *NTN1* can be considered an oncogene [54]. On the other hand, *MYH10* acts as a tumor suppressor and its downregulation is associated with poor outcomes in hepatocellular carcinoma [55]. The exact role of these genes in MM requires further studies. Similarly, in BTZ-resistant cells, we observed hypermethylation in the *ARHGAP26* gene, which is involved in tumorigenesis and progression of human cancers. However, in various types of cancer, its expression changes significantly [29]. For instance, Qian et al. demonstrated that *ARHGAP26* hypermethylation resulted in reduced gene expression, which may be an early event in the pathogenesis of AML. Similar associations were observed by Bojesen and colleagues [56,57]. On the other hand, Li et al. showed an increase in its expression in prostate cancer [58]. Our observations of the global methylation profile in BTZ-resistant MM cells and VD-incubated control cells are consistent with the trend shown by other researchers that hypomethylation is associated with cancer development and progression [59,60,61]. On the contrary, it can be hypothesized that hypermethylation suggests a decreased proliferative potential of malignant cells and a better response to therapy. The results of our preclinical study in an in vitro model revealed intriguing relationships and associations that should be further investigated and provided the background for subsequent clinical trials in MM.

## 5. Conclusions

VD and VK have the potential to induce epigenetic changes in MM cells. Moreover, these alterations are associated with the proliferative potential of plasma cells as well as the development of resistance to BTZ. Globally, hypermethylation is correlated with decreased proliferative potential of malignant plasma cells, and hypomethylation with BTZ resistance. There is an urgent need to conduct in vivo studies in clinical trials to determine whether similar relationships will also occur in patients with multiple myeloma. If the results turn out to be consistent, it will be necessary to introduce new supplementation recommendations as part of adjuvant therapy.

## 6. Study Limitations

Although our study provided novel insights into the role of VD and VK2 MK7 in MM and shed more light on the regulation of epigenetic mechanisms, it has several drawbacks. First, our study was conducted on only one MM cell line. Second, our study provided preliminary results with the intention to shed more light on the pathophysiology of BTZ-resistance and potential easy-to-implement and cost-effective adjuvant therapies. Nevertheless, it should be emphasized that the obtained results require further validation in more complex preclinical models, e.g., using different MM cell lines, especially those obtained from MM patients and other second-generation proteasome inhibitors (PIs) such as carfilzomib and ixazomib. Ultimately, the results obtained should be evaluated in an animal model, and finally, in a clinical trial.

## Figures and Tables

**Table 1:** Primer sequences used in the qRT-PCR reactions.

Gene	Primer Sequences
*ARHGAP26*	F 5′-CCTTAGGGGCAGAGTTGCTC-3′ R 5′-CAGCCCCGATCTGTTCCTTT-3′
*MYH10*	F 5′-CCAAACGTCAGGGAGCATCT-3′ R 5′-GGTGGCTCATGAAGACCAGA-3′
*PMP2*	F 5′-AAGCTCTGGGTGTGGGGTTA-3′ R 5′-TGCAGGGTTACGATGCTCTT-3′
*RFX8*	F 5′-AGCAGCTCCATGTCACACAG-3′ R 5′-TCCTTGACTTGCTTGAGCGT-3′
*BAMBI*	F 5′-GGTGCAGGAGCTGACTTCTT-3′ R 5′-ATTCCAGCTCCCTTGGATGC-3′
*CLEC12b*	F 5′-AAAAGAGGGCATCCAGCTCC-3′ R 5′-GCCCAGTTGCTGGGATAAGT-3′

**Table 2:** Real-time quantitation of selected genes in the U266 cell line.

Gene	Control_3 Mean ± SD; 95% CI	BTZ_3 Mean ± SD; 95% CI	D_3 Mean ± SD; 95% CI	DK_3 Mean ± SD; 95% CI	BTZ_DK_3 Mean ± SD; 95% CI
*ARHGAP26*	1.00 ± 0.08; 0.75–1.25	0.78 ± 0.09; 0.50–1.06	1.16 ± 0.01; 1.01–1.31	1.34 ± 0.11; 1.00–1.68	1.69 ± 0.24; 0.94–2.43
*MYH10*	0.21 ± 0.03; −0.28–0.71	0.06 ± 0.01; 0.01–0.10	0.09 ± 0.02; 0.03–0.16	0.18 ± 0.07; −0.06–0.42	0.19 ± 0.04; 0.06–0.32
*PMP2*	0.05 ± 0.02; −0.01–0.12	0.11 ± 0.04; −0.02–0.23	0.36 ± 0.24; −0.39–1.11	0.41 ± 0.03; 0.30–0.52	0.44 ± 0.17; −0.09–0.98
*RFX8*	0.23 ± 0.04; 0.10–0.35	0.13 ± 0.03; 0.02–0.25	0.40 ± 0.12; 0.02–0.78	0.73 ± 0.16; 0.21–1.25	0.33 ± 0.13; −0.09–0.75
*BAMBI*	11.50 ± 1.94; 5.57–17.42	11.55 ± 1.41; 7.26–15.84	15.46 ± 1.37; 10.98–19.95	29.10 ± 7.02; 7.73–50.46	20.58 ± 4.66; 6.39–34.77
*CLEC12b*	0.16 ± 0.09; −0.15–0.46	0.10 ± 0.01; 0.04–0.15	0.22 ± 0.07; −0.02–0.45	0.38 ± 0.1; 0.06–0.69	0.35 ± 0.07; 0.12–0.58

SD—standard deviation, 95% CI—95% confidence interval of the mean (D—treated with vitamin 25(OH)D3; BTZ—treated with bortezomib; DK—treated simultaneously with vitamin 25(OH)D3 and K2MK7; BTZ_DK—treated simultaneously with bortezomib, vitamin 25(OH)D3, and K2MK7; Control—control group; 3—third incubation).

## Data Availability

The datasets used and analyzed during the current study are available from the corresponding author on reasonable request.

## Supplementary Materials

The following supporting information can be downloaded at: https://www.mdpi.com/article/10.3390/nu16010142/s1, Figure S1: Methylation profile after second subsequent BTZ, VD, and VK treatments of U266 myeloma cells; Figure S2: Methylation profile after second VD treatments of U266 myeloma cells.; Figure S3: Methylation profile after second VD and VK treatments of U266 myeloma cells.
