# Supplementary material for: Vitamin D and K Supplementation Is Associated with Changes in the Methylation Profile of U266-Multiple Myeloma Cells, Influencing the Proliferative Potential and Resistance to Bortezomib
Source: Nutrients. 2023 Dec 31;16(1):142. doi: 10.3390/nu16010142 (PMC10780809; doi:10.3390/nu16010142)
Supplement: Supplementary file 1 [file nutrients-16-00142-s001.zip › descriptions for supplementary.pdf]

Figure S1

Methylation profile after second subsequent BTZ, VD and VK treatments of U266 myeloma cells. A, B) charts show differences in methylation levels in U266 cells treated with BTZ, VD and VK relative to cells treated with BTZ alone ($p<0.05$); C) View of changes in the level of methylation in individual chromosomes. Orange represents hypomethylation and green represents hypermethylation ($p<0.05$); D) Classification of differentially methylated sites in the genome according to their location relative to the CpG islands ($p<0.05$); E) Classification of differentially methylated sites in the genome according to their location relative to the transcription start site (TSS) ($p<0.05$). (VD—treated with vitamin 25(OH)D3; BTZ—treated with bortezomib; VD_VK—treated simultaneously with vitamin 25(OH)D3 and K2MK7; BTZ_VD_VK—treated simultaneously with bortezomib, vitamin 25(OH)D3 and K2MK7; Control - control group; 2 – second incubation).

Figure S2

Methylation profile after second VD treatments of U266 myeloma cells. A, B) charts show differences in methylation levels in U266 cells treated with VD relative to untreated control cells ($p<0.05$); C) View of changes in the level of methylation in individual chromosomes. Orange represents hypomethylation and green represents hypermethylation ($p<0.05$); D) Classification of differentially methylated sites in the genome according to their location relative to the CpG islands ($p<0.05$); E) Classification of differentially methylated sites in the genome according to their location relative to the transcription start site (TSS) ($p<0.05$). (VD—treated with vitamin 25(OH)D3; Control - control group; 2 – second incubation).

Figure S3

Methylation profile after second VD and VK treatments of U266 myeloma cells. A, B) charts show differences in methylation levels in U266 cells treated with VD and VK compared to untreated control cells ($p<0.05$); C) View of changes in the level of methylation in individual chromosomes. Orange represents hypomethylation and green represents hypermethylation ($p<0.05$); D) Classification of differentially methylated sites in the genome according to their location relative to the CpG islands ($p<0.05$); E) Classification of differentially methylated sites in the genome according to their location relative to the transcription start site (TSS) ($p<0.05$). (VD_VK—treated simultaneously with vitamin 25(OH)D3 and K2MK7; Control - control group; 2 – second incubation).
