# Supplementary figures and images for: Vitamin D and K Supplementation Is Associated with Changes in the Methylation Profile of U266-Multiple Myeloma Cells, Influencing the Proliferative Potential and Resistance to Bortezomib
Source: Nutrients. 2023 Dec 31;16(1):142. doi: 10.3390/nu16010142 (PMC10780809; doi:10.3390/nu16010142)

A

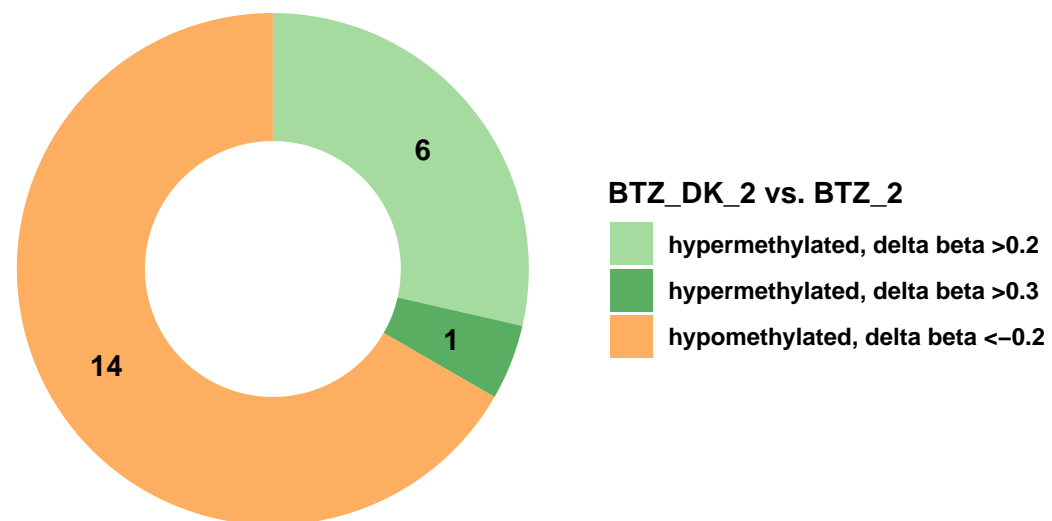

B

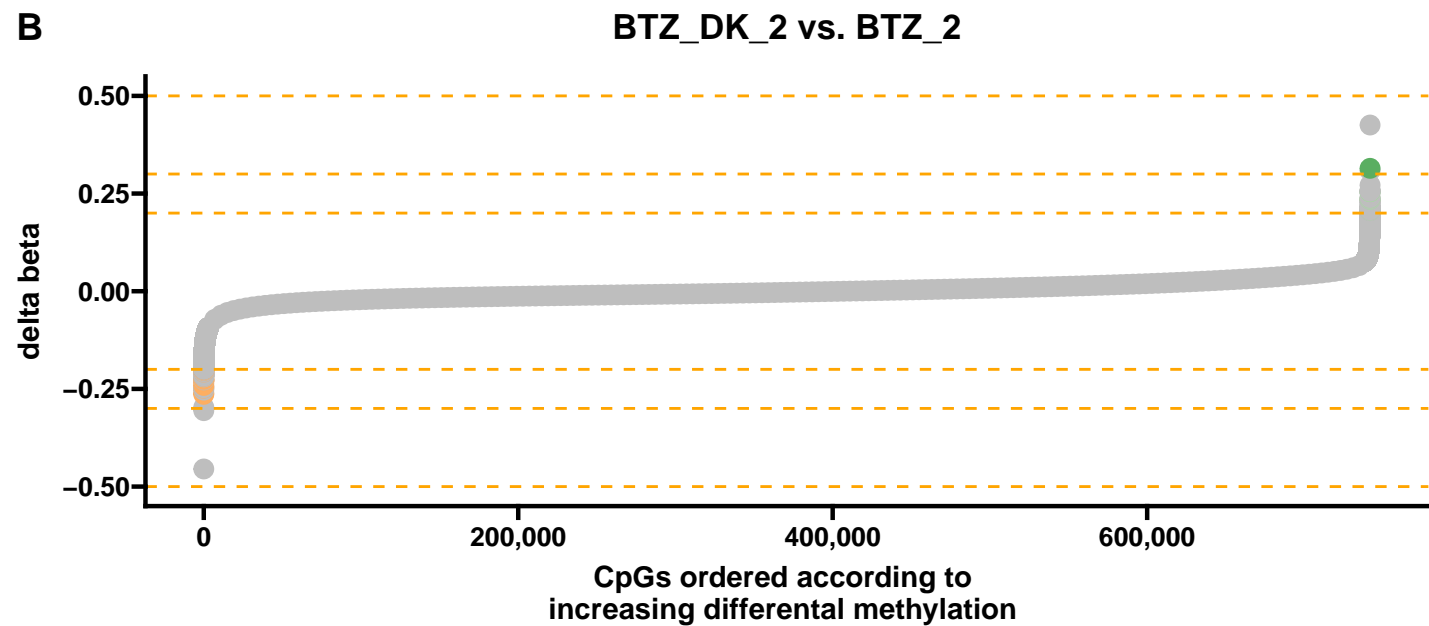

C

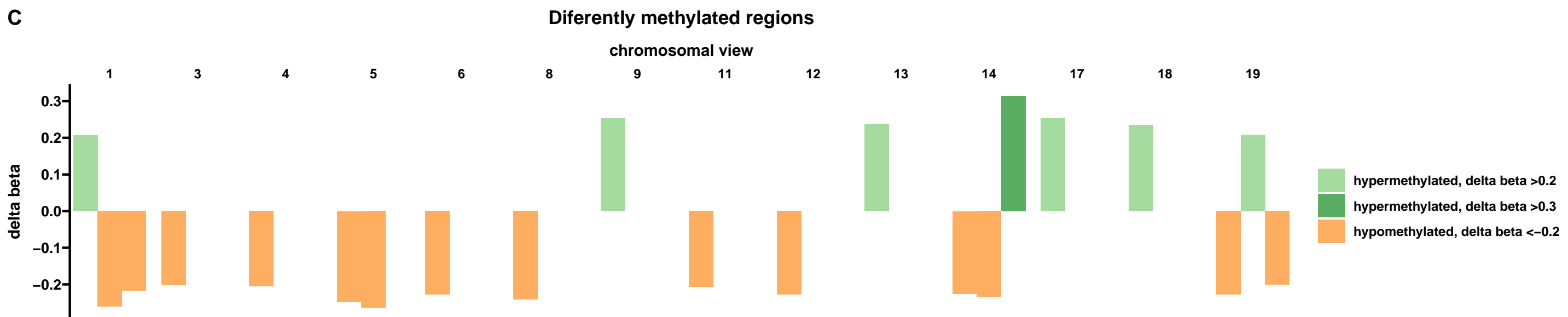

D Relative to CpG islands

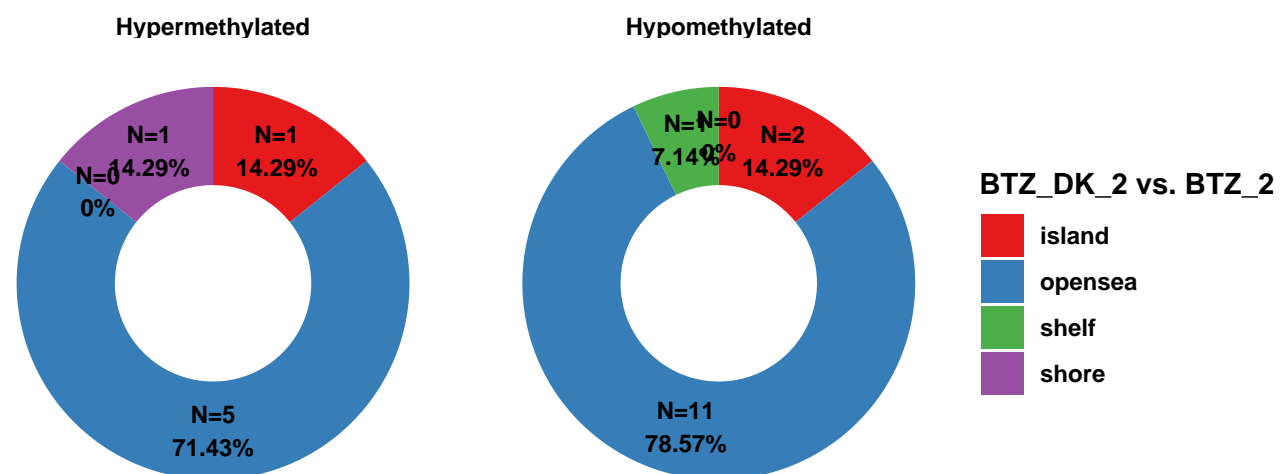

E Relative to TSS

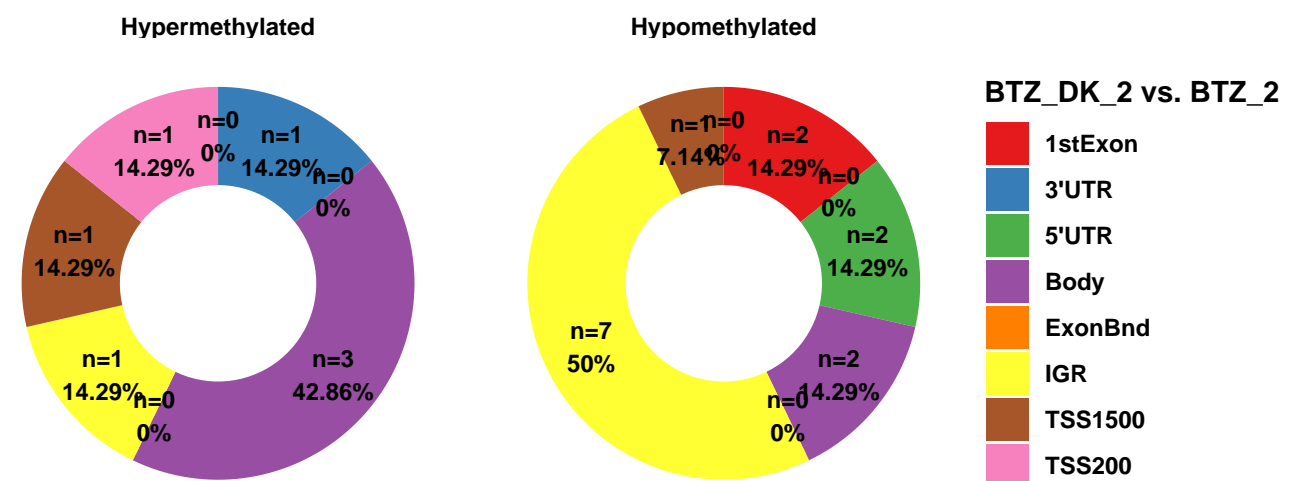

Supplement: Supplementary file 1 [file nutrients-16-00142-s001.zip › Figure S1.pdf]

A

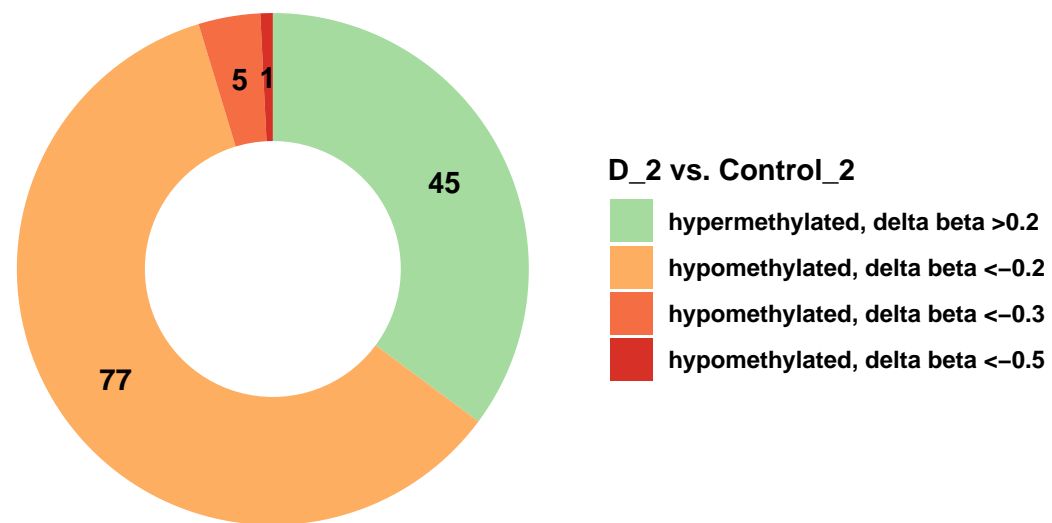

B

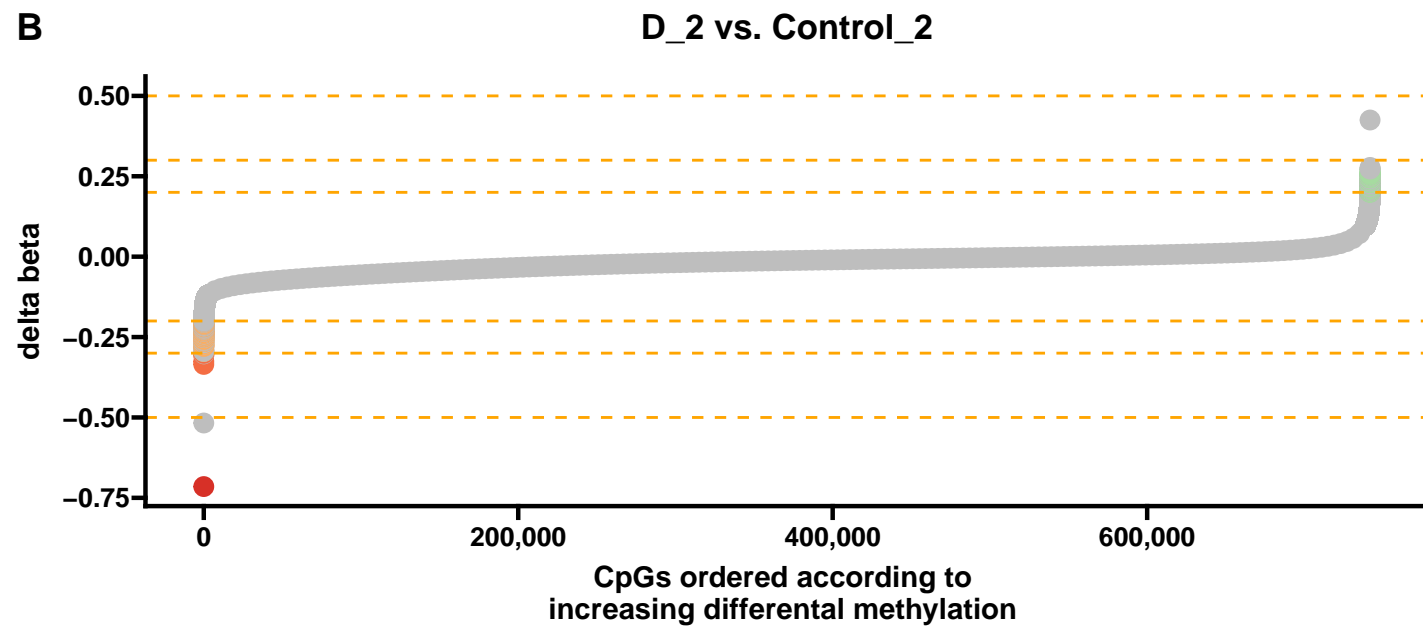

C

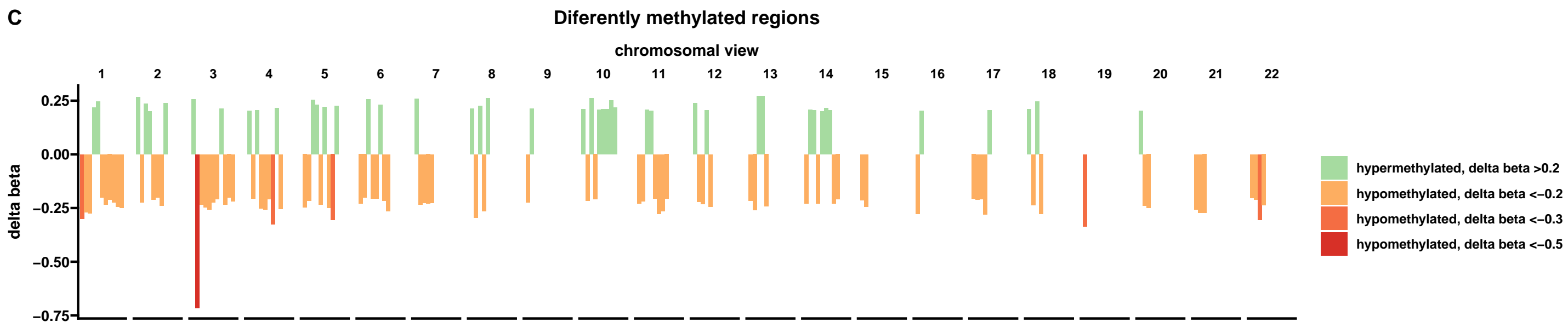

D

Relative to CpG islands

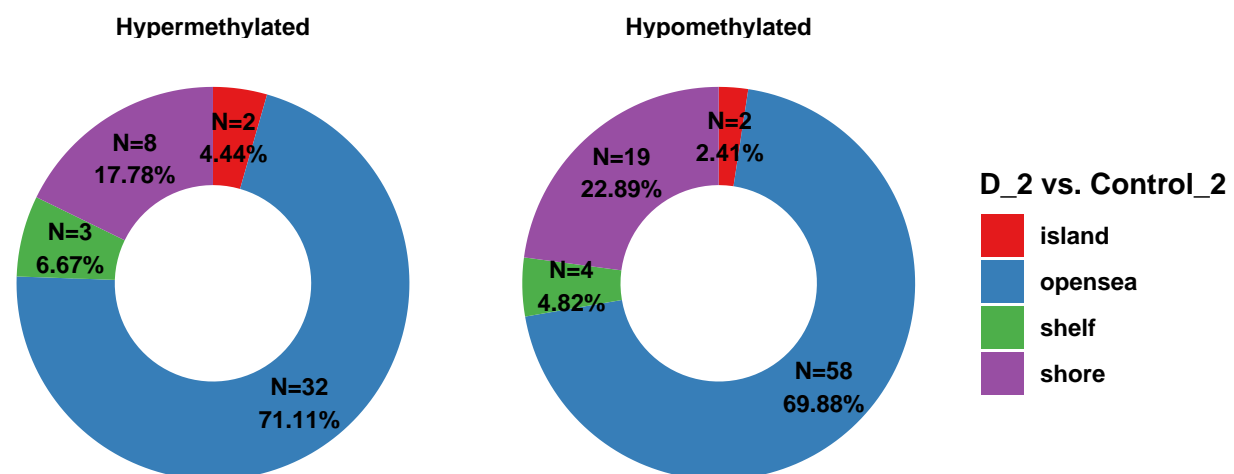

E

Relative to TSS

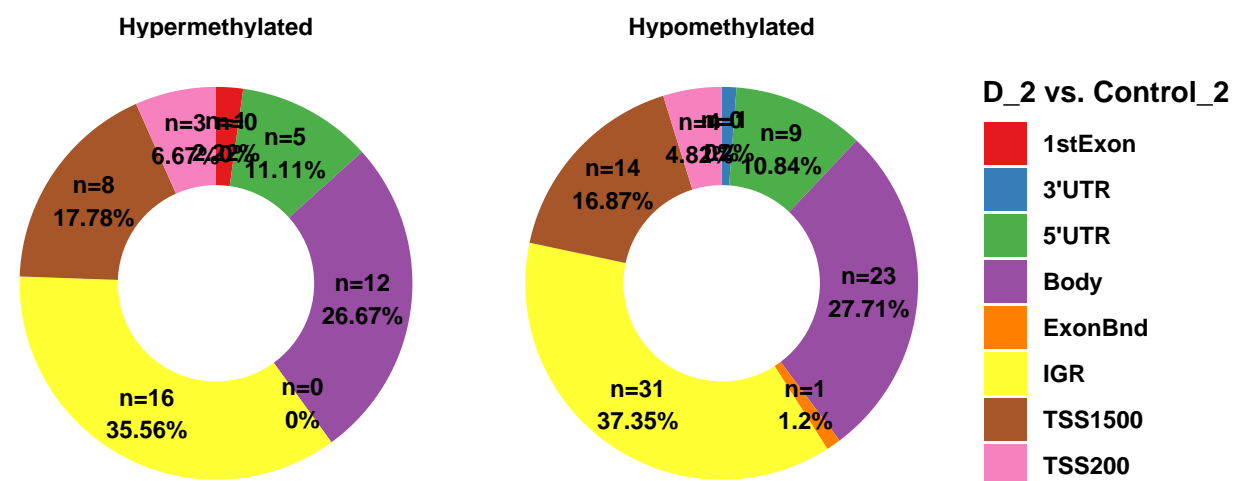

Supplement: Supplementary file 1 [file nutrients-16-00142-s001.zip › Figure S2.pdf]

A

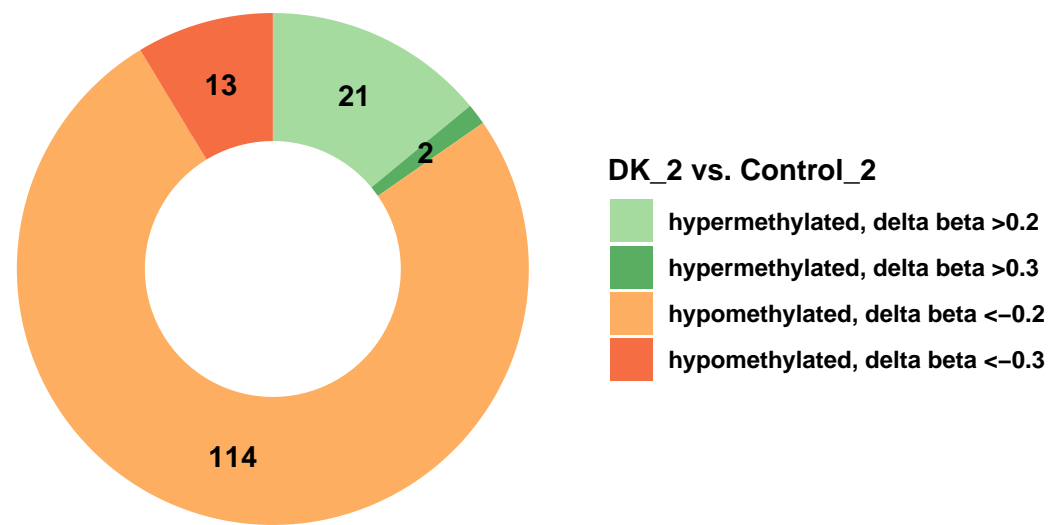

B

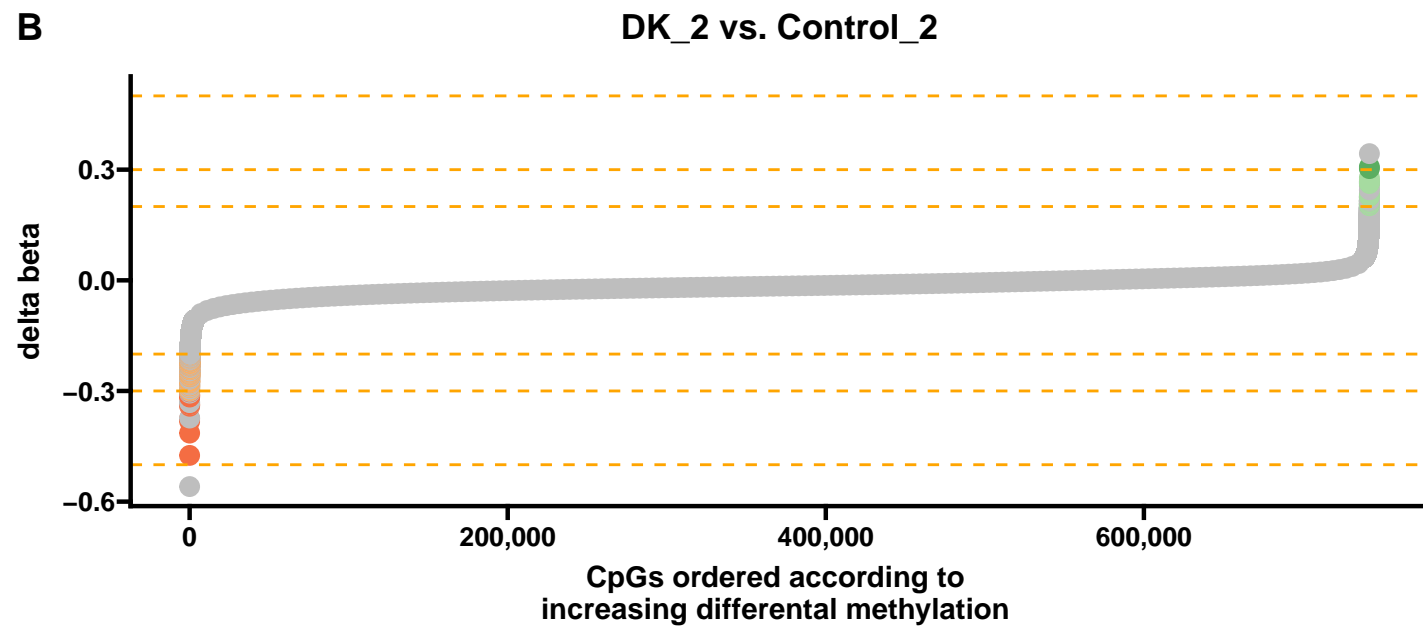

C

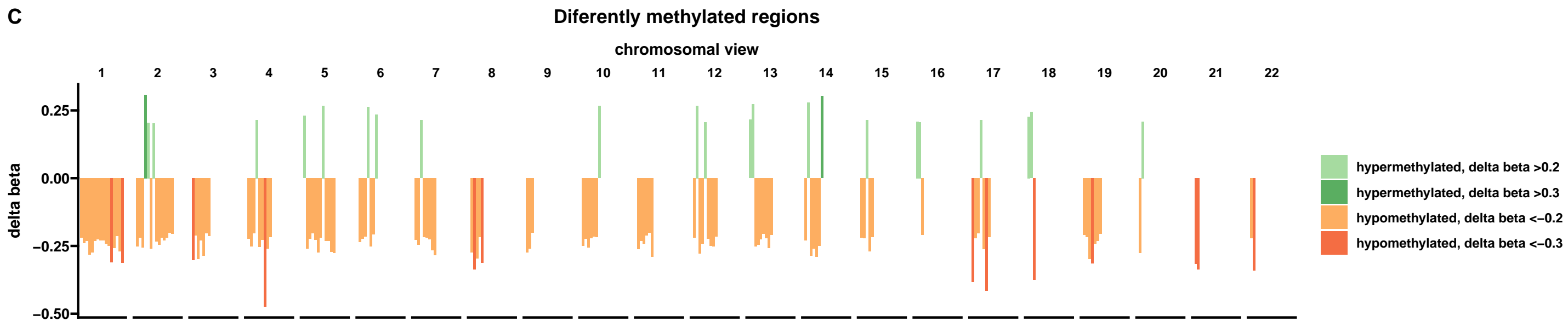

D

Relative to CpG islands

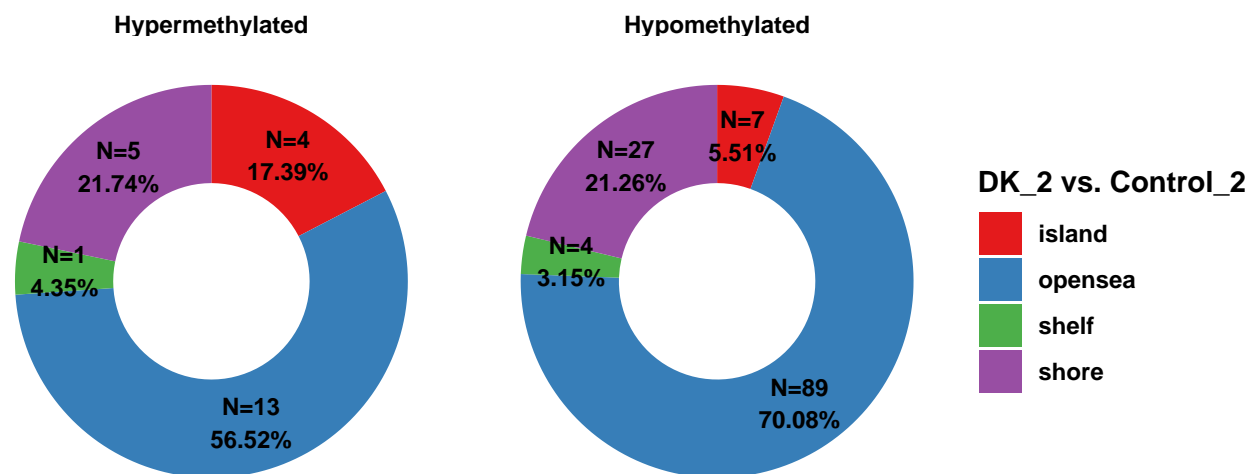

E

Relative to TSS

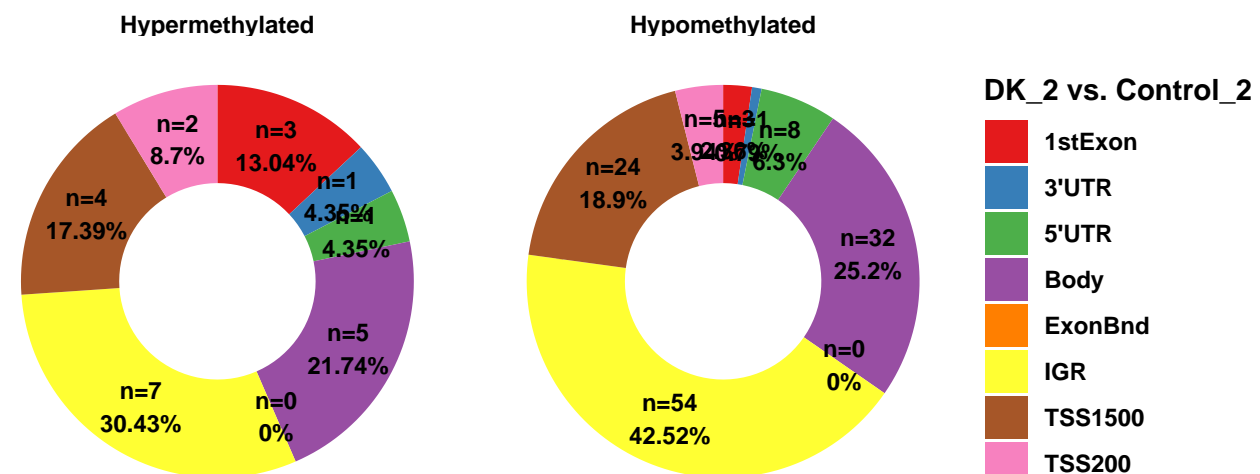

Supplement: Supplementary file 1 [file nutrients-16-00142-s001.zip › Figure S3.pdf]
